# A versatile strategy for rapid conditional genome engineering using *loxP* sites in a small synthetic intron in *Plasmodium falciparum*

**DOI:** 10.1038/srep21800

**Published:** 2016-02-19

**Authors:** Matthew L. Jones, Sujaan Das, Hugo Belda, Christine R. Collins, Michael J. Blackman, Moritz Treeck

**Affiliations:** 1 Moritz Treeck Laboratory, The Francis Crick Institute, Mill Hill Laboratory, The Ridgeway, London NW71AA, United Kingdom; 2 Michael J. Blackman Laboratory, The Francis Crick Institute, Mill Hill Laboratory, The Ridgeway, London NW71AA, United Kingdom

## Abstract

Conditional genome engineering in the human malaria pathogen *Plasmodium falciparum* remains highly challenging. Here we describe a strategy for facile and rapid functional analysis of genes using an approach based on the Cre/lox system and tailored for organisms with short and few introns. Our method allows the conditional, site-specific removal of genomic sequences of essential and non-essential genes by placing *loxP* sites into a short synthetic intron to produce a module (loxPint) can be placed anywhere in open reading frames without compromising protein expression. When duplicated, the loxPint module serves as an intragenic recombineering point that can be used for the fusion of gene elements to reporters or the conditional introduction of point mutations. We demonstrate the robustness and versatility of the system by targeting the *P. falciparum* merozoite surface protein 1 gene (*msp1*), which has previously proven refractory to genetic interrogation, and the parasite exported kinase FIKK10.1.

Protein function can be conditionally interrogated by systems that allow exogenous control of mRNA abundance, stability, protein turnover or localization. While these are useful tools, they often deliver only partial depletion of a gene product, leading to outcomes that can be difficult to interpret. Of the tools available to conditionally silence a gene, methods that allow conditional DNA deletion are often preferred as these completely remove the targeted gene or important parts thereof. One of the most widely used conditional DNA deletion methods relies on Cre recombinase-mediated recombination between two 34-nucleotide *loxP* sequences. To examine gene function using the Cre/lox method, *loxP* sites are introduced at positions that either flank an entire gene of interest or that lie within introns, allowing Cre-driven deletion of an entire gene or key exon^1^. Both of these options are however not suitable for species such as *P. falciparum* where few genes have introns and where poor transfection efficiency precludes facile introduction of multiple *loxP* sites. In addition, the *P. falciparum* genome is highly AT-rich (>80% in intergenic regions) making targeted genome modifications in these regions complicated. Finally, currently used conditional genome recombination methods in *Plasmodium*^2^,^3^ do not allow conditional domain rearrangements, point mutations, or directed fusion to heterologous sequences such as epitope tags or reporters. While a range of important human and animal pathogens such as *Trypanosomes*, several fungal species and model organisms like yeast and *Dictyostelium* contain few and short introns, no strategy has yet been developed to rapidly place *loxP* sites into intronless genes.

To substantially increase the ability to conditionally and rapidly modify genes with the potential for broad use in species that contain few introns or many mono-exonic genes, an ideal system would allow: 1) a single transfection step for target-directed gene manipulation; 2) the introduction of “silent” *loxP* sequences anywhere in the genome, including within exon sequences; 3) the conditional rearrangement of specific genetic elements such that one stretch of DNA can be rapidly replaced by another for use in domain swapping, introduction of point mutations, epitope tagging or to generate a reporter for successful recombination events; and 4) tight temporal control of Cre activity that allows rapid recombination rates regardless of the organism used.

Here we have devised a simple strategy that meets all of the above criteria. We validate its use in the human malaria parasite *Plasmodium falciparum*, an organism that presents many inherent difficulties in genetic modification. We use a standardized small module (loxPint) that consists of a short, *loxP* site-containing intron that can replace endogenous introns or can be placed in open reading frames of episomes as well as in chromosomal genes. If duplicated, the loxPint module is reconstituted after Cre-mediated recombination, enabling the fusion, replacement, or specific deletion of flanking genetic elements. To achieve rapid recombination rates we use parasite lines stably expressing a chemically-regulated Cre (DiCre)^1^,^2^. We demonstrate that this system allows the rapid, conditional control and modification of essential and non-essential genes alike.

## Results

### Introduction of silent *loxP* sites using a standardized intron

**Figure 1:**
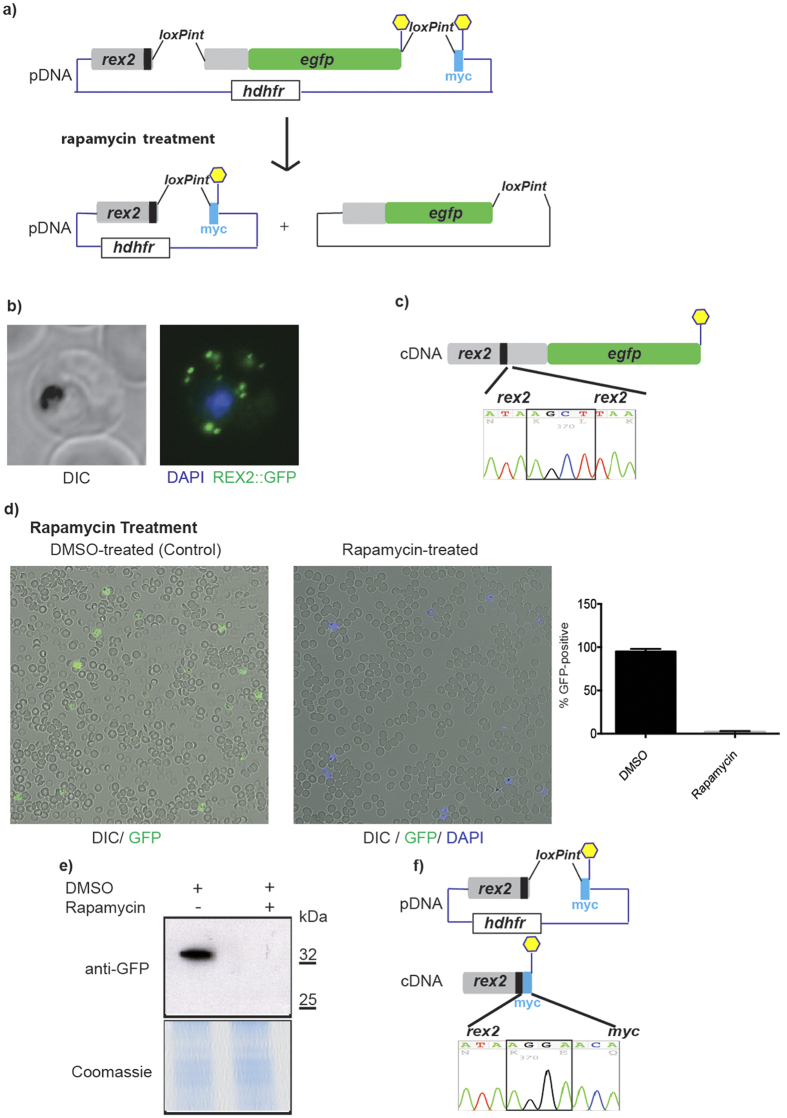
A *loxP* containing synthetic intron loxPint allows inducible, targeted DNA rearrangement. (**a**) Schematic shows the loxPint module in the *rex2* gene (black rectangle represents the native transmembrane domain). Stop codons are represented as yellow hexagons. A second loxPint is located downstream of the GFP coding sequence followed by a myc epitope coding sequence. Rapamycin treatment causes dimerization of two Cre-recombinase subunits in the DiCre-expressing 1G5DC *P. falciparum* line. Cre-driven LoxP-site recombination between the loxPint modules reconstitutes loxPint and places the myc coding sequence in frame with *rex2*. (**b**) Live fluorescence image showing *pRex2:loxPint:GFP* confirms Rex2.GFP expression and correct targeting to the host cell. (**c**) LoxPint is appropriately spliced. The chromatogram shows the nucleotides identified in sequencing reactions from cDNA from Rex2:loxPint:GFP parasites. Black box represents exon-exon boundaries. The resulting amino acid translation is shown in light grey. LoxPint is absent from sequenced cDNA and the correct *rex2* coding sequence is present, confirming correct splicing. (**d**) RAP-induced Cre-mediated DNA excision reduces Rex2.GFP expression. GFP positive parasites are reduced by 97.6% (+/−1.1%) upon rapamycin treatment (error bars are SD). (**e**) Western blot showing RAP-induced DNA excision results in the loss of Rex2.GFP expression. (**f**) LoxPint is reconstituted and correctly spliced after Cre-mediated recombination. Schematic shows the *pRex2:loxPint:GFP* construct after Cre-mediated excision before (pDNA) and after (cDNA) splicing. The 5′ *rex2* sequence that remains after Cre-driven DNA excision is transcribed in-frame with the myc epitope coding sequence as shown by cDNA sequencing. The black box in the chromatogram highlights the exon-exon boundary.

Limitations of novel genetic tools in *P. falciparum* are mainly dictated by its low transfection efficiency. For that reason, facile application of the Cre/lox system^2^, while introducing a tightly controllable conditional genetic system into the parasite, has been hampered by the fact that flanking target genes with *loxP* sites can take many months. Such manipulations also carry the risk that introduction of *loxP* sites into flanking regulatory sequences can interfere with gene expression levels. In other organisms, *loxP* sites are commonly placed in introns where they are unlikely to interfere with promoter function. However, introns are rare in the *P. falciparum* genome (45.5% of all protein coding genes are single-exon genes, 24.0% contain 2 exons and only 30.5% contain 3 or more exons) (www.plasmoDB.org), which limits the use of that strategy. We reasoned that placement of *loxP* sites into a small intron that naturally occurs in the *P. falciparum* genome might allow it to be used as a universal module that could be placed into exons, enabling the flanking of critical segments of genes with silent *loxP* sites in a single transfection step. Ideally this intron would be short and lack long repetitive A or T stretches because these often cause substantial problems with gene synthesis. We screened the *P. falciparum* genome and identified an intron of the *sera2* gene (PlasmoDB ID PF3D7_0207900) which meets these criteria. To test whether the *sera2* intron can accommodate a *loxP* site, we generated a proof-of-principle episomal expression construct, *pRex2:loxPint:gfp*, in which the intron of *rex2* was replaced with the *sera2* intron containing an integral *loxP* site (loxPint, sequence shown in supplemental table 2) and then fused in frame with the green fluorescent protein gene (*gfp)* (Fig. 1a). We chose REX2 because of its small size and because it has been well characterized as a GFP fusion protein that is efficiently exported from the intracellular parasite into the host erythrocyte^4^. We reasoned this would allow its use as a reporter for correct loxPint splicing, even for exported proteins. This is an important property as other conditional methods that have been applied in *P. falciparum*, such as protein degradation systems, are not suitable for the study of genes encoding exported or secreted proteins^5^. A second loxPint module was placed downstream of the *gfp* open reading frame, followed by a myc epitope tag sequence. Cre-mediated recombination between the two *loxP* sites was predicted to reconstitute the loxPint module, excising the C-terminal region of REX2 and the entire GFP sequence, replacing this sequence with that encoding the myc epitope tag. Activation of Cre recombinase activity in the 1G5DC *P. falciparum* clone is induced by rapamycin (RAP), which mediates rapid heterodimerisation of the constitutively-expressed DiCre polypeptides^2^. This was predicted to lead to recombination between the two *loxP* sites and excision of the 3′ end of *rex2* and *gfp* (Fig. 1a). We transfected *pRex2:loxPint:gfp* into 1G5DC parasites and confirmed faithful expression and localization of REX2.GFP (Fig. 1b). To test whether loxPint was correctly spliced in transfected parasite lines, we analysed plasmid DNA (pDNA) and reverse transcribed DNA (cDNA) of *rex2:loxPint:gfp* parasites. Two species were amplified from cDNA, one likely corresponding to the spliced variant, and one slightly higher running band that suggested incomplete splicing of the loxPint. Sanger sequencing confirmed that the lower band corresponded to the correctly spliced version of *rex2:loxPint:gfp* and verified correct retention of the loxPint following splicing (Fig. 1c). These results showed that the loxPint module allows functional splicing in a heterologous locus.

### The loxPint module allows conditional domain fusions in an episomal context

To test for correct DiCre-mediated recombination between the two loxPint sequences in *pRex2:loxPint:gfp*, we treated transfected parasites with RAP for 4 h then analysed excision between the two loxPint modules by evaluating GFP expression. As expected, RAP-treated but not control parasites, showed near complete loss of GFP expression as measured by live microscopy (Fig. 1d) and Western Blot (Fig. 1e). To test whether RAP treatment had reconstituted the loxPint module in a form that was still correctly spliced, we examined the parasites by immunofluorescence analysis (IFA) with anti-myc antibodies. Unfortunately, we could not confirm a specific signal in RAP-treated parasites because the anti-myc antibodies cross-reacted strongly with other parasite antigens on IFA and the truncated REX2:myc protein was likely too small to be detected on Western blot. However, sequencing of cDNA from the RAP-treated parasites confirmed correct splicing of the reconstituted loxPint module, effectively fusing the myc tag in frame with the 3′ end of the remaining *rex2* open reading frame (Fig. 1f). This shows that duplication of the loxPint module allows directed, conditional fusion of distinct protein-coding sequences.

### The loxPint module can be used in an endogenous genomic locus and is quantitatively spliced

**Figure 2:**
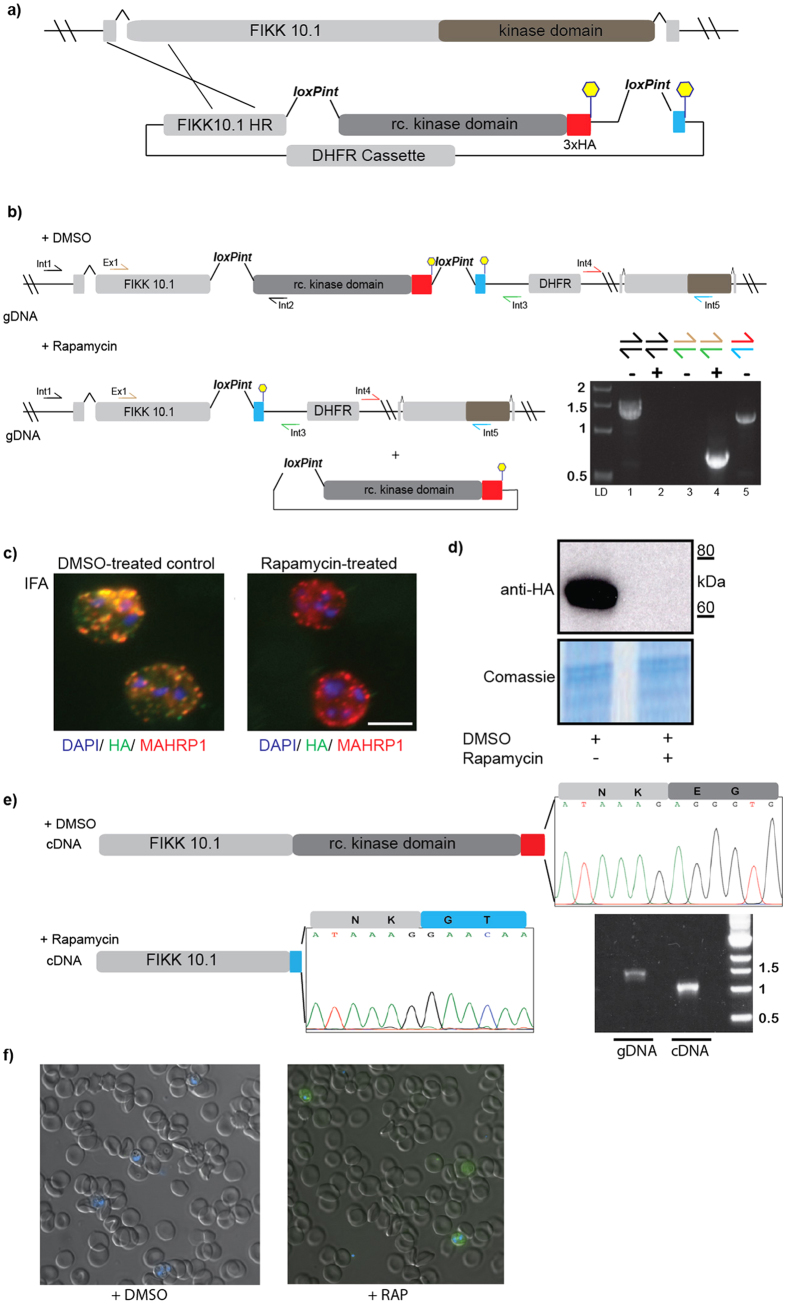
Use of the loxPint strategy allows the silent targeting of the exported kinase FIKK10.1 and conditional domain fusion. (**a**) Overview of the strategy to introduce two loxPint modules in a single transfection step using single homologous recombination at the 3′ end of FIKK10.1. (**b**) Schematic of the structure of the *FIKK10.1* locus after integration of construct *pfikk10.1:loxPint:HA,* which introduces loxPint upstream of a recodonized FIKK10.1 kinase domain. The loxPint is duplicated downstream of an HA3 epitope tag sequence to allow removal of the HA3-tagged FIKK10.1 kinase domain by Cre-driven DNA excision. (**c,d**) The introduced loxPint does not interfere with FIKK10.1 expression or export to the host erythrocyte as shown by IFA and Western Blot. Cre-driven DNA excision induced by treatment with RAP results in loss of the FIKK10.1.HA3 signal in IFA and Western Blot. (**e**) cDNA sequencing results from DMSO and RAP treated FIKK10.1 loxPint parasites shows that the loxPint is correctly spliced and RAP treatment leads to reconstitution of the loxPint module, allowing conditional domain re-arrangements. PCR results show complete splicing of the loxPint in FIKK10.1 in cDNA but not gDNA. (f) Live fluorescence imaging showing GFP expression in the DMSO vs. RAP treated *pfikk10.1:loxPint:HA:gfp* parasite line.

Encouraged by these results, we decided to further validate our method by attempting to introduce a loxPint module into the open reading frame of a chromosomal parasite gene with the aim to tag the gene of interest in the same gene modification step as the introduction of two repeated loxPints. For this we chose to investigate the gene encoding an uncharacterized parasite kinase called FIKK10.1 (PF3D7_1016400) that is predicted to be exported into the host cell^6^. The *fikk10.1* gene includes two short introns close to its 5′ and 3′ ends and encodes a protein with a C-terminal kinase domain, allowing targeting by 3′ replacement. We generated a targeting construct, *pfikk10.1:loxPint:HA*, designed to introduce a recodonised *fikk10.1* kinase domain into the endogenous locus flanked by loxPint modules (Fig. 2a) and transfected this construct into 1G5DC parasites. Integration of *pfikk10.1:loxPint:HA* was expected to result in expression of an HA-tagged chimeric FIKK10.1 under the control of its endogenous promotor (Fig. 2b). Correct integration of the construct and expression of the modified gene was confirmed by PCR, IFA, and Western Blot (Fig. 2b–d). As predicted, treatment of this engineered parasite line with RAP led to recombination between the loxPint modules flanking the recodonized *fikk10.1* kinase domain and subsequent loss of the entire kinase domain and HA tag (amino acid residues 251–641 of FIKK10.1.HA (Fig. 2c,d)). Sequencing of cDNA from DMSO-treated control and RAP-treated parasites confirmed correct splicing of the loxPint both before and after DiCre-driven DNA rearrangement (Fig. 2e). Because we had observed incomplete splicing from the episomally expressed *rex2:loxPint:gfp* we tested splicing efficiency of the loxPint in the context of the *fikk10.1* locus. A single PCR product was obtained from gDNA and cDNA, corresponding to the unspliced and spliced versions respectively, indicating complete splicing of the loxPint (Fig. 2e).

To further verify the use of the loxPint module for conditional domain fusions we generated the parasite line FIKK10.1:loxPint:HA:GFP which shares all the features of FIKK10.1:loxPint:HA except that RAP treatment leads to fusion of the first 250 amino acids of FIKK10.1 with GFP. As expected, treatment of this parasite line with RAP led to replacement of the kinase domain of FIKK10.1 with GFP (Fig. 2f). No effects on parasite growth were observed after RAP treatment, indicating that FIKK10.1 is likely a non-essential kinase under the conditions tested here and further work will be required to identify the biological function of FIKK10.1. However, collectively these data show that the loxPint module can serve as a recombination point for rapid, efficient conditional deletion or fusion of DNA elements.

### The loxPint module can be placed in essential genes and does not compromise expression levels

**Figure 3:**
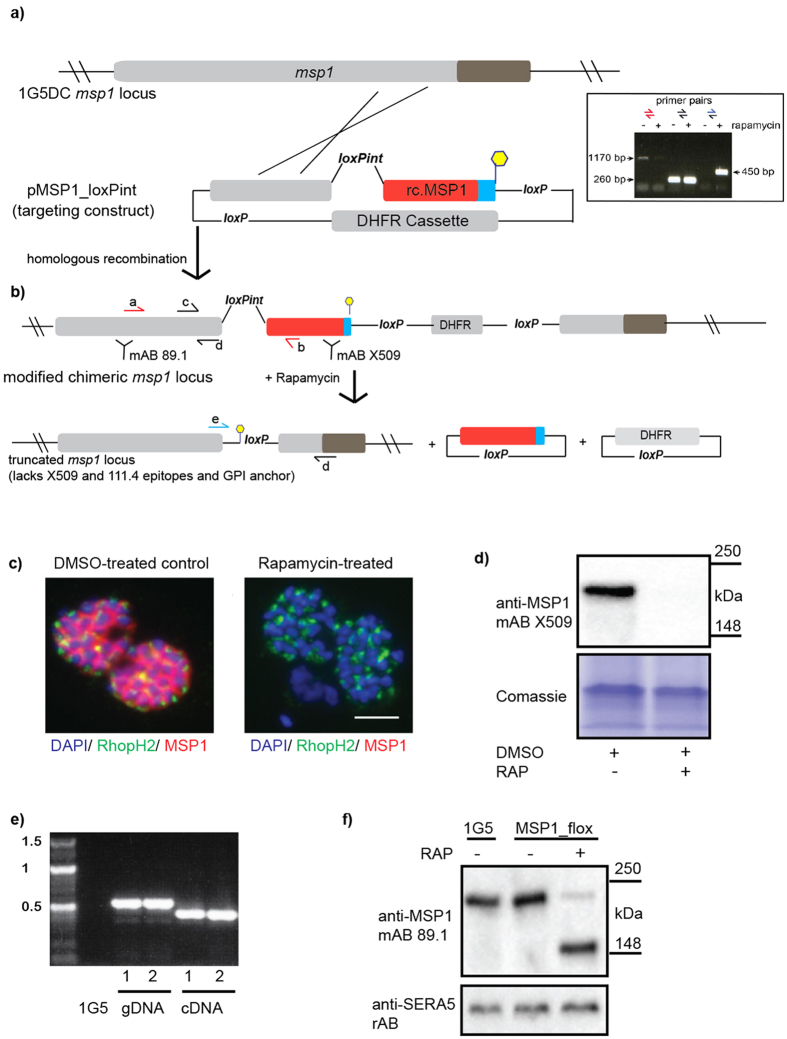
Use of the loxPint strategy allows the silent targeting of *msp1* in *P. falciparum*. (**a**) Schematic showing the *P. falciparum* 1G5DC *msp1* locus following the introduction of loxPint by homologous recombination via a 3′ replacement. To force integration downstream of the loxPint module, recodonized MSP1 sequence is used downstream of that module (red). The endogenous C-terminal end is exchanged with a polymorphic MSP1 variant specifically recognized by mAb 111.4, thus effectively epitope tagging the gene. The modified *msp1:loxPint* locus is followed by a second *loxP* site that allows removal of the *msp1 3*′ coding sequence. The *msp1* GPI anchor coding sequence is shown in blue. Yellow hexagon represents a stop codon. Correct integration of the construct into the endogenous locus is verified by PCR (insert and primer binding sites shown in (**b**)). (**b**) Cre-driven DNA excision results in the truncation of MSP1 (loss of amino acid residues 1240–1682, which includes the mAb X509 and mAb 111.4 epitopes). (**c**) The introduced loxPint is efficiently spliced and does not interfere with MSP1 expression or localisation, as shown by normal expression of MSP1 in control (DMSO-treated) schizonts by IFA. MSP1 was detected by IFA with mAb X509. Cre-driven DNA excision induced by treatment with RAP results in loss of the mAb X509 epitope. The rhoptry marker anti-RhopH2 mAb 61.3 (green)was used as a control. Schizont nuclei are stained with DAPI (blue). Scale bar, 5 μM. (**d**) Western blot, showing that reactivity with mAb X509 is lost upon RAP-treatment. (**e**) PCR showing complete splicing of the loxPint module in *msp1:loxPint* in two independent preparations of cDNA. (**f**) Western blot showing that introduction of the loxPint module into the *msp1* locus does not alter MSP1 expression levels compared to the parent 1G5 parasite line. Excision results in expression of a truncated MSP1 that is still recognised by mAb 89.1, which binds an epitope within the N-terminal part of MSP1 (see (**b**)).

To test the effectiveness of the loxPint in the modification of a proven essential genomic gene we tested its utility in modification of the single exon gene encoding merozoite surface protein-1^7^ (*msp1,* PlasmoDB ID PF3D7_0930300). Using single-crossover homologous recombination, a loxPint module was incorporated into the *msp1* coding sequence upstream of the glycosyl phosphatidyl inositol (GPI) anchoring signal that tethers this protein to the surface of the merozoite, the invasive blood-stage form of the parasite. In the same gene modification step, a second *loxP* site was introduced downstream of the *msp1* stop codon (Fig. 3a). Excision of the *loxP*-flanked sequence was predicted to produce a truncated form of MSP1 lacking its C-terminus and GPI anchor (amino acid residues 1277–1720). The construct readily integrated into the endogenous *msp1* locus of transfected 1G5DC parasites (Fig. 3a). This was confirmed by IFA and Western blot analysis (Fig. 3c,d). RAP-treatment of the engineered parasites resulted in the expected deletion of the MSP1 C-terminal domain and GPI anchor (Fig. 3b), as confirmed using monoclonal antibodies specific for the deleted C-terminal region of the protein (Fig. 3c,d). As we recently reported, RAP treated parasites showed a severe defect in egress from the host erythrocyte^8^. In tests designed to assess the splicing efficiency of the *msp1* gene containing the loxPint, a single ~600 bp band corresponding to completely spliced transcript was amplified from cDNA, showing complete splicing of the transcript (Fig. 3e). This was confirmed by Sanger sequencing (data not shown). To test that overall expression levels of MSP1 were not compromised by the introduction of the loxPint module, we analysed parental 1G5DC and MSP1_loxPint parasites by Western blot using monoclonal antibodies specific for MSP1. This detected no significant alterations in MSP1 expression levels in msp1:loxPint parasites compared to the parental 1G5DC line (Fig. 3f). These results confirmed that the loxPint can be readily introduced into open reading frames of essential, mono-exonic endogenous genes and is efficiently spliced without impacting on gene expression levels.

## Discussion

In this study we have demonstrated that a small, readily synthesised genetic module comprising a *loxP* site integrated into an intron can be introduced into a range of genomic loci to: (1) replace native introns; (2) inserted into genomic exons; and (3) can even be incorporated into non-native recodonised sequences in the malaria parasite *Plasmodium falciparum*. In all cases the module is correctly and efficiently spliced. Compared to currently employed strategies for conditional gene deletion, our system represents a substantial step forward as it allows the conditional fusion and removal of DNA, irrespective of the presence of endogenous introns, in a single transfection step. Simultaneous 3′ tagging of a gene in the same modification step as the introduction of loxPint modules provides an invaluable tool for the study of uncharacterized genes, as shown for the parasite kinase FIKK10.1. In that case loss of the C-terminal HA tag served as a reporter for efficient recombination between the two loxPint modules and GFP was used as a positive reporter for efficient recombination. Importantly, the ability to exchange one stretch of DNA with another will allow conditional introduction of point mutations in the future (see Supplementary Fig. 1). Here we used single cross-over homologous recombination to introduce the loxPint module into the *P. falciparum* genome, but it is worth noting that successful introduction of an artificial exon flanked by two loxPint sequences for internal domain deletion has recently been achieved in *P. falciparum* using CRISPR/Cas9 technology^9^,^10^ (Emma Sherling, Michael Blackman and Christiaan van Ooij unpublished results (see also Supplementary Fig. S1). It is important to stress that while CRIPSR/Cas9 technology allows precise insertion of loxPint modules into genomic loci, it does not on its own allow the functional interrogation of essential genes.

The ability to conditionally disrupt genes such as FIKK10.1 that are non-essential in cell culture is just as important as it is for essential genes. Many genes that are not required during the blood stages in cell culture are readily lost. As such, phenotypes associated with gene knock-out studies that aim to interrogate for example sexual stage formation, bear a substantial risk of gene loss during the gene modification step. Our strategy, which allows the incorporation of 2 *loxP* sites in a single transfection step and delete a genetic element within a single parasite generation (~48 h) in a RAP controlled manner significantly reduces the time that it takes to generate such mutants by conventional methods, including CRIPSR/Cas9. The observation that the loxPint introduced into the non-essential FIKK10.1 parasite line is quantitatively spliced is important since it shows that selective pressure is not required for correct splicing of the loxPint and so it can be used for essential and non-essential genes alike. While we did not detect unspliced loxPint in the two genomic loci tested here, the presence of unspliced product from the REX2:GFP episome indicates that under some circumstances splicing may not be 100% efficient. Whether this is due in this case to the mRNA originating from an episome with potentially incomplete 5′ and or 3′ regulatory mRNA elements is unknown. However, given that we have shown that the loxPint works faithfully in two distinct endogenous loci, we are confident that it will prove a versatile novel tool to interrogate the many unknown and known essential and non-essential genes in the human malaria parasite.

The combination of a standardized small intron bearing a *loxP* site in conjunction with DiCre will open up its use to species beyond the malaria parasite where splicing machinery is present. While a strategy has been previously developed in mice where a synthetic intron was used to introduce a cassette (conditionals by inversion (COIN- module)) this is unlikely to be functional in organisms with fewer and shorter introns^11^,^12^ because of its large size. In conclusion, the method we present here is a major breakthrough in our ability to study the function of essential and non-essential genes in the malaria parasite *P. falciparum*. Because of the simplicity of its design we predict it will prove widely useful.

## Materials and Methods

### *P. falciparum* culture and transfection

*P. falciparum* clone 1G5DC^2^ and the transfected parasite lines established here were cultured as described^13^. Routine synchronization was by Percoll enrichment and sorbitol treatment. For transfection, either purified schizont-stage or ring-stage parasites (>5% parasitemia) were electroporated with 50–150 ug of plasmid DNA as described^2^,^14^,^15^,^16^ and selection was performed with 5 nM WR99210 (Jacobus Pharmaceuticals). To select for plasmid integration into the *fikk10.1* or *msp1* locus, transfectants were grown in the absence and then presence of 5 nM WR99210 (21 days in the absence of drug followed by growth in the presence of drug until a viable parasite population was re-established). Cycling between growth in the absence and then presence of WR99210 was performed at least twice. Plasmid integration into the *fikk10.1 or msp1* locus was verified by PCR using the primers described in Supplementary Table 1. For *fikk10.1* modification, integration was confirmed after each drug cycle.

To induce DiCre-driven *loxP* site recombination, synchronized ring-stage parasites were treated with 100 nM RAP (Sigma) or DMSO only (final concentration 1% v/v) for 4 h. Parasites were subsequently washed twice with warm RPMI and returned to culture. Samples used for nucleic acid extraction were taken at least 24 h after rapamycin treatment, and samples used for immunofluorescence (IFA) or protein extraction were taken at the end of the same asexual cycle (~44 h following RAP treatment) or in subsequent cycles.

### loxPint design and Construct Assembly

To identify an intron that could be used to silently introduce *loxP* sequences, we used a combination of manual searching and motif screening (using the search term “AGGTAA.{30,120}AGAT” with the PlasmoDB motif search feature, which incorporates bioinformatics analysis of *P. falciparum* 3D7 intron structure performed by Zhang *et al.*^17^. This allowed the identification of several short intron sequences that could potentially be used to introduce *loxP* sites. We chose the short intron of *sera2* because it contained few extended mono-nucleotide tracts and could readily be synthesized by gene synthesis services. The 34 nt *loxP* sequence was inserted into the *sera2* intron after identification of its probable branch point^17^ so that splicing would likely proceed correctly even in the presence of these additional nucleotides. See Supplemental Table 2 for the complete *Sera2Intron:LoxP* sequence.

To generate the loxP:intron proof-of-principle construct *pRex2:loxPint:gfp*, we replaced the Rex2.GFP cassette of a pARL-based Rex2.GFP-encoding plasmid^4^ (a kind gift of Tobias Spielmann, Bernhard Nocht Institute for Tropical Medicine, Hamburg, Germany) with a *rex2.GFP* sequence in which the endogenous *rex2* intron was replaced with the loxPint sequence. The GFP-coding sequence was followed by a stop codon and a second loxPint, all followed by a myc-Tag and stop codon. This sequence was synthesized (Geneart®, see also Supplementary Table 2) then amplified using primers Rex2.POP.F and Rex2.POP.R and cloned into KpnI and AvrII-digested pARL:rex2GFP using Gibson assembly^18^.

To generate pMSP1_loxPint, the 998 bp *msp1* targeting fragment was amplified from *P. falciparum* 3D7 genomic DNA using primers endo3D7-MSP1-BglII-targ-F and endo3D7-MSP1-R. The recodonized fragment was created by amplifying recodonized sequence from plasmid pZ-3D7-MSP138/42 (a kind gift of Dr Christian Epp, University of Heidelberg, Germany) using primers syn3D7-MSP1-F and syn3D7-MSP1 + PstI-R. The fragments were then joined together by overlapping PCR (using primers endo3D7-MSP1-BglII-targ-F and syn3D7-MSP1 + PstI-R) to create a fragment with 5′ BglII and 3′ PstI restriction site overhangs. This was ligated into the pHH1-MSP1_19_ backbone, which has the wMSP1_19_ in the 3′ end (created by digesting the plasmid pMSP1chimWT^19^ with BglII and PstI). The resultant plasmid construct was called pHH1-3D7wt. To introduce the loxPint, a ~400 bp sequence corresponding to the loxPint fragment flanked by *msp1* targeting sequence at the 5′ and 3′ends (-CCTCAACC**AG**-loxPint-**AT**GTAACTCC-; bold letters indicate a naturally occurring AGAT motif, which effectively serves as the intron-exon boundary) was synthesised by Geneart® (see also Supplementary Table 2) and introduced into pHH1-3D7wt using restriction sites HpaI and BstEII. This generated plasmid pMSP1_loxPint. The plasmid structure was confirmed by nucleotide sequencing on both strands. See Supplementary Table 2 for the full recodonized *msp1* fragment sequence.

The FIKK10.1 targeting construct was generated by removal of the loxPint in the rex2:loxPint:gfp cassette of *pRex2:loxPint:gfp* using inverse PCR. This also introduced a point mutation into *rex2:GFP* that prevents REX2.GFP expression. The resulting plasmid was digested with Not1 to introduce the *fikk10.1* homology region and recodonized kinase domain by Gibson assembly to generate the plasmid *pfikk10.1:loxPint:HA*. The *fikk10.*1 homology region consists of *fikk10.*1 nucleotides 1 to 1033 and was amplified using primer FK10.1HRF and FK10.1HRR. The recodonized kinase domain corresponds to *fikk10.1* nucleotides 1034 to 2319 lacking the 3′ native intron. The recodonized kinase domain was synthesized (Geneart®, see also Supplementary Table 2) and then amplified using primers FK10.1RCF and FK10.1RCR. The final plasmid was sequenced to confirm correct assembly. See Supplementary Table 2 for the full recodonized *fikk10.1* kinase domain sequence including the added loxPint sequence.

All primers used in this study can be found in Supplementary Table 1.

### Immunofluorescence

Live microscopy: Rex2:GFP and FIKK10.1loxPint:GFP expression was quantified by imaging live synchronized parasites that had been treated with RAP or DMSO for 4 h in at least the previous cycle. Parasites were taken fresh out of cell culture and treated with 4′,6-diamidino-2-phenylindole (DAPI) to visualize nuclei. Images were taken using a Ti-E Nikon microscope using a 40x and 100x TIRF objective at room temperature equipped with an LED-illumination and an Orca-Flash4 camera. Images were processed with Nikon Elements software.

For immunofluorescence of the FIKK10.1loxPint parasite line, air-dried blood films were fixed for 4 min in ice-cold methanol and subsequently rehydrated in PBS for 5 min. Slides were blocked in 3% (w/v) bovine serum albumin (BSA) in PBS containing kanamycin (50 μg/ml) for 1 h and subsequently incubated with primary or secondary antibodies in 3% (w/v) BSA in PBS containing kanamycin (50 μg/ml) for 45 min. Antibody concentrations used were: rat anti-HA high affinity (Roche) (1:1000), rabbit anti-MAHRPI ((1:1000) a kind gift of Lindsay Parish and Julian Rayner). Images were taken using a Ti-E Nikon microscope using a 100x TIRF objective at room temperature equipped with an LED-illumination and an Orca-Flash4 camera. Images were processed with Nikon Elements software.

For MSP1 immunofluorescence, thin films of *P. falciparum* cultures were air dried, fixed in 4% paraformaldehyde (in PBS) for 30 min at room temperature and cell membranes permeabilised in 0.1% (v/v) Triton X-100 (SIGMA) for 10 min. Fixed slides were then washed three times with PBS for 10 min and blocked overnight at 4 °C in 3% (w/v) BSA in PBS. Slides were probed with anti-MSP1 mAb X509^20^ using undiluted supernatant from culture hybridomas. Slides were also probed with anti-RhopH2 mAb 61.3 (1:250)^21^. Slides were probed with primary sera for 30 min at 37 °C and then washed for 10 min in PBS. Slides were then probed with an appropriate fluorescent secondary antibody (1:2000) and washed for 10 min with PBS. Slides were stained with DAPI and mounted in PBS/glycerol and images collected using AxioVision 3.1 software on an Axioplan 2 Imaging system (Zeiss) using a Plan-APOCHROMAT 1006/1.4 oil immersion objective^22^.

### Western Blotting

Western Blotting was performed according to standard methods. Briefly, Rex2.GFP or FIKK10.1.HA-expressing parasites were released from erythrocytes by addition of 0.1% (w/v) saponin/PBS for 5 min at room temperature. 0.1% saponin lysates were centrifuged (>15000 × G) and the resulting parasite pellet was solubilized with 1X sample buffer with 5% beta-mercaptoethanol at a concentration of 2.5–5 × 10^8^ parasites/ml. Parasite extracts were subjected to SDS PAGE and transferred onto nitrocellulose membranes. Rex2.GFP was visualized by probing the blots with rabbit anti-GFP antibodies (Abcam (1:500)) and goat anti-rabbit-HRP (Insight Biotechnology (1:4000)). FIKK10.1.HA was visualised using rat anti-HA hi affinity (Roche (1:500)) and goat anti-rat-HRP (1:4000 (Sigma)). For detection of MSP1, schizonts were Percoll-enriched according to standard methods then solubilized by addition of SDS sample buffer, subjected to SDS PAGE, and proteins transferred to nitrocellulose membranes. MSP1 was visualized by probing blots with anti-MSP1 mAb X509 or mAB 89.1^20^ (undiluted hybridoma culture supernatant) and a goat anti-human-HRP secondary antibody (Sigma). SERA5 was visualized using polyclonal rabbit anti-SERA5 serum.

### Nucleic acid extraction, cDNA synthesis, and sequencing

DNA was extracted from *P. falciparum* parasite lines using the QIAamp DNA Blood MiniKit (Qiagen). RNA was extracted as described^23^ and DNA was removed using the Ambion TURBO DNA-free kit (Applied Biosystems). cDNA was produced from isolated, DNAse-treated RNA by first-strand cDNA synthesis using random hexamers (Superscript III cDNA synthesis kit, Invitrogen). For sequencing, primers allowing PCR amplification across the LoxPint module both before and after DiCre-induced recombination were designed for *Rex2, MSP1,* and *FIKK10.1* and the resulting PCR fragment was either sequenced directly or blunt cloned into pCR.Blunt II-TOPO using a Zero Blunt PCR cloning kit (Invitrogen).

## Additional Information

**How to cite this article**: Jones, M. L. *et al.* A versatile strategy for rapid conditional genome engineering using *loxP* sites in a small synthetic intron in *Plasmodium falciparum*. *Sci. Rep.* **6**, 21800; doi: 10.1038/srep21800 (2016).

## Supplementary Material

Supplementary Information
