# Supplementary material for: A versatile strategy for rapid conditional genome engineering using loxP sites in a small synthetic intron in Plasmodium falciparum
Source: Sci Rep. 2016 Feb 19;6:21800. doi: 10.1038/srep21800 (PMC4759600; doi:10.1038/srep21800)
Supplement: Supplementary Information [file srep21800-s1.pdf]

A versatile strategy for rapid conditional genome engineering using loxP sites in a small synthetic intron in *Plasmodium falciparum*

Matthew L. Jones, Sujaan Das, Hugo Belda, Christine R. Collins,
Michael J. Blackman, Moritz Treeck

Supplementary Figure 1

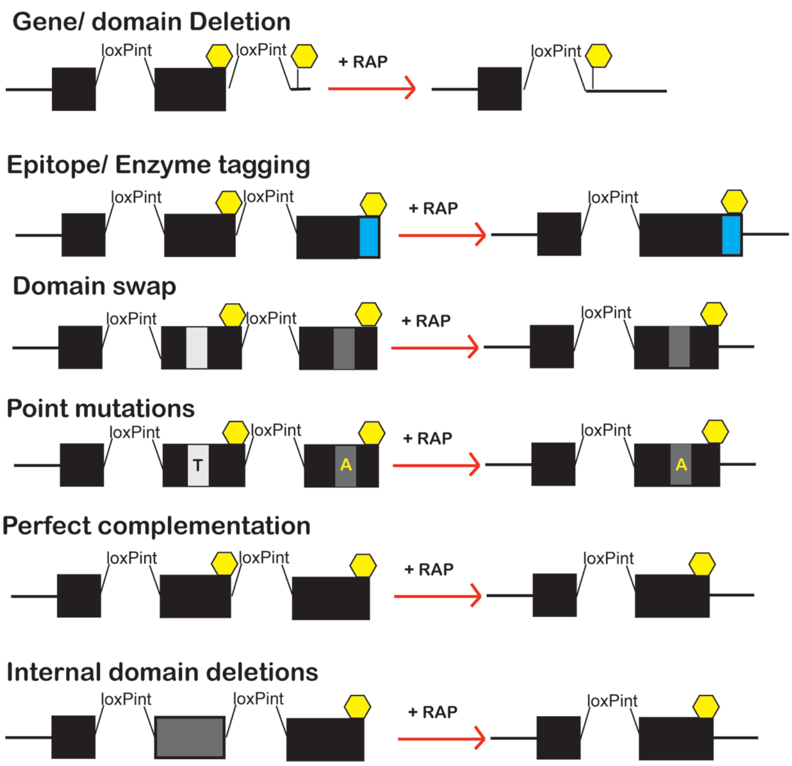

Supplemental Figure 1:
Possible strategies for conditional genome engineering using the loxPint module.
The reconstitution of the loxPint module after RAP treatment allows the conditional fusion or deletion of domains. This opens new avenues for conditional epitope or enzyme tagging (for example with biotin ligase A (BirA)), conditional domain swaps, introduction of point mutations or perfect complementation, that is, replacing a genetic element with an identical copy. Using CRIPSR the introduction of two loxPint modules into an open reading frame to delete only parts of a gene will be feasible. Yellow hexagons represent stop codons.

Used For:	Primer Name:	Primer Sequence:
pRex2:loxPint:gfp	Rex2.POP.F	GTTTTTTTTAATTTCTTACATATAACTCGACCCCGGGATGGTACCTTTATGAAAATGTATTTAGCTG
	Rex2.POP.R	GAAAAACGAACATTAAGCTGCCATATCCCCGCGGCTGCAGTTACAGATCCTCTTCTGAG
pfikk10.1:loxPint:HA	FK10.1HRF	GCCAAGCTATTTAGGTGACACTATAGAATACTCGCGGCCGCATGACTCTTATTAATAGAAGTTATGTTTTATTTGG
	FK10.1HRR	GCTATACGAAGTTATTGTATATTATTTTTTTTATTTACCTTTATTATAACCATGTGTAGGTATAGAAGTTAATTTC
	FK10.1RCF	GAAATTAACTTCTATACCTACACATGGTTATAATAAAGGTAAATAAAAAAAATAATATACAATAACTTCGTATAGC
	FK10.1RCR	CTACTAAGATCTCCTCCTAAGTCTGTTACGTTAGCGGCCGCTTAGACCGCATAATCCGGTACATCGTATGGATACG
pMSP1_loxPint	syn3D7-MSP1-F	GTGGTAGTTCAGGATCCACAAAAGAAGAAACCC
	syn3D7-MSP1+PstI-R	GCATGTCCTGCAGCTTGCCCTCTATGAGCTTTGATATGATGG
	endo3D7-MSP1-BglII-targ-F	CCAACAAAGATCTGCATCCTCTACCAATACCC
	syn3D7-MSP1+PstI-R	GCATGTCCTGCAGCTTGCCCTCTATGAGCTTTGATATGATGG
pfikk10.1:loxPint:HA integration confimation	Int1	GTCCTTCATTAATTTGATGGTCA
	Int2	CACATAGTTTTCTCCGCACAGCACGTATTCGC
	Int3	CAACATACACATTTTTACAGTTATAAATACAATCAATTG
	Int4	CCCCAGGCTTTACACTTTATGCTTCCGGCTC
	Int5	CTTAATAAATAATCCTACTCTATCACTACCATCTC
pMSP1_loxPint integration confirmation	MSP1-UOT-FOR	GGAACATCATCTACATCCAGTCCTGG
	REV3	GTAGAGATCCTGATGTGGGGATC
	FOR1	CCATTTCTACAACAGAGATGG
	REV4	GCATTTTGTCTTGGCCAAGTTC
	P2 FOR	GTAAATAAAAAAAATAATATACAATAACTTCGTATAGCATACATTATACGAAGTTAT

Supplemental Table 1:
Contains all primer sequences used in this study.

loxPint sequence	gtaaataaaaaaaataatatacaATAACTTCGTATAGCATACATTATACGAAGTTATtatatatgtatatatatatatatttatatattttatattcttttag
**Used For:**	**Synthesized Sequence**
pRex2:loxPint:gfp	cccgggatggtacctttatgaaaatgtatttagctgaaatttttagttctggtaaagagtctttgttatctttaaaggatactttaggatctagtaatttttctccactgaaaccatgtgaaggccttgagtgtttaccacaagtattatttttgtacgttatat<br>ttcttttattatgtacaggaatttttatgcataataagaataaggtaaataaaaaaaaataatatacaataacttcgtatagcatacattatacgaagttattatatatgtatatatatatatatttatatattttatattcttttagcttaaaaaacaacaaa<br>ggttaacaaatatgaatgattcatatgatgcaaaaaaattcaggattcgcagaacagtatgatcaacaacaacatcctaggatgagtaaaggagaagaacttttcactggagttgtcccaattcttgttgaattagatggtgatgttaatgggcacaaa<br>ttttctgtcagtggagagggtgaaggtgatgcaacatacggaaaacttacccttaaatttatttgcactactggaaaactaccagttccatggccaacacttgtcactactttcgcgtatggtcttcaatgctttgcgagatacccagatcatatgaaaca<br>gcatgactttttcaagagtgccatgcccgaaggttatgtacaggaaagaactatatttttcaaagatgacgggaactacaagacacgtgctgaagtcaagtttgaaggtgatacccttgttaatagaatcgagttaaaaggtattgattttaaagaag<br>atggaaacattcttggacacaaattggaatacaactataactcacacaatgtatacatcatggcagacaaacaaaagaatggaatcaaagttaacttcaaaattagacacaacattgaagatggaagcgttcaactagcagaccattatcaacaa<br>aatactccaattggcgatggccctgtccttttaccagacaaccattacctgtccacacaatctgccctttcgaaagatcccaacgaaaagagagaccacatggtccttcttgagtttgtaacagctgctgggattacacatggcatggatgagctctac<br>aaataactcgaggtaaataaaaaaaaataatatacaataacttcgtatagcatacattatacgaagttattatatatgtatatatatatatatttatatattttatattcttttaggaacaaaaactcatctcagaagaggatctgtaactgcagccgcgg
pfikk10.1:loxPint:HA	gtaaataaaaaaaataatatacaataacttcgtatagcatacattatacgaagttattatatatgtatatatatatatatttatatattttatattcttttagagggtggtcgtgtgcaggaaatgttcaagacctttgtctcatccaaagaaggcgatggc<br>agtgatcgtgttgggctgtttattaaaaaaattcctgtggatatttgggtgaaacagttcgaactgatgaatgcgtacaatggcgaatacgtgctgtgcggagaaaactatgtgatggaagcgaccacactggcgtttttgaacgaatactatccgggc<br>attactccgaaactgtataaaattctttacgagccggagaagaaagagtatgatattgagcagaataccccggactgtatgtttcatgacctgaatgtctttaatgacattcttagcgcgcgtctcaaatgtaacatgaatgggtacattgttatcatta<br>gcgagctgtttggcgaagatatctacacttatctgacgaaacagaaaaaaaaaaatattttcgcgcttcactcgtacatgaaacggaaaaaaattctctttgaatgtctgaacgtgttgcggaaactgcatgacgcaggcttatgccaccttgatatct<br>caccccagaatattcttatgtcctataactttgaaattcgtctgtgcgatttggccaaatccacccccatctatacaaataacttacgccatctgaaaaacatgaatggtacgtatttgttcgagagctgtgtgccaacgattggcaaaattcgctccatg<br>ccaccagaatgctgggaaatttcgcgtaaatatattcgtatgaagatttgtgagccactggaacaactgtcccctatcaccgacctggacgaacgtgcgcctttctatttcgatgtcacctcagcggataagtttatgttaggcgtgctgtttatttggat<br>ctggaacaacgattacttgtggaagagcagcgacattgaacaggatattgactttttgcgcgtctcggaatgcgatatggatattgatgttttcgaactgactcgtacgtggccttatgaactcaaaaagattattcaaaaactcctgcagaccgaagg<br>tcggaaaaacctcaacctccatgaattatgcgcgcatccctggtggttttctaaaatgatgtacccatatgatgtaccggattacgcaggttatccgtatgacgttccggactatgctggctcgtatccatacgatgtaccggattatgcggtctaa
pMSP1_loxPint	atgtccagcacatcgtcacctggcaacacgacagtaaataccgcccagtctgctacacactccaactcccagaaccagcagagcaacgcttctagcaccaacacccagaatggggtagcagttagtagcggccctgctgtggtggaggaatcgcat<br>gaccccctcactgtattatctatttcaaacgacctaaaagggattgtgtccctcctcaatttaggtaataagaccaaggtccctaacccccttgactatcagcactacggaaatggagaagttttatgaaaacatcctgaagaacaacgacacctatttt<br>aacgacgacataaagcagttcgtgaagagtaacagtaaagtgattaccgggctgacagaaacccagaaaaatgctttaaatgatgagatcaagaaactgaaagacacactccagctctccttcgatctgtacaacaagtacaaactaaagctgga<br>cagattattcaataagaagaaggagcttgggcaagataagatgcagattaagaagctaactttactgaaggagcagctcgagagcaagctcaactccctgaataatccacataatgtgctccagaacttttccgtattcttcaataagaagaaagaa<br>gcagagattgccgagacggaaaataccctcgaaaacactaagatattactgaaacactataaagggctggtgaagtattacaacggagagtctagcccattgaagactctttcagaagtgtcaattcaaaccgaggataactacgcaaacctaga<br>aaagttcagagtgctgagcaaaatcgacggcaaactcaatgataacctacacctcggaaaaaaaaaagctgagcttcctgtccagtggacttcatcatttaattaccgaattgaaagaagttatcaaaaacaaaaactacactgggaacagcccatc<br>tgaaaataataaaaaggtcaacgaggccctcaagtcttatgaaaatttccttccagaagcaaaagtgacaaccgtcgtgacccccccccagcccgatgtcacccccagccctctaagcgtgagagtgtctggatcaagtggctccacaaaagaaga<br>aacccagatccccacatcaggatctctactgaccgagttgcagcaggtcgtccaactccagaattatgacgaggaagacgacagcctcgtggttttgccaatcttcggcgaatcagaagacaacgacgagtacctagaccaagtggtcaccgggga<br>agcgattagtgtcactatggacaatatcctcagcggcttcgagaacgagtatgacgtgatctacctcaaaccactagccggagtttacagaagtctcaagaagcagatcgaaaagaacatcttcacctttaatctaaacctaaacgacatcttgaatt<br>cccggctgaaaaagcggaaatacttcctcgacgtactggagtcggatttgatgcagtttaagcacatctccagcaacgaatacattatcgaggactcgttcaaactgttaaactccgagcagaagaacaccctgctgaagtcctacaaatatatcaa<br>agagtcagtcgagaacgatattaaattcgcccaagaaggcataagctactacgaaaaggtcctcgccaaatacaaggacgatctggagtctatcaaaaaggtcatcaaagaagagaaagagaaatttcccagttctcccccctacaacgccgccct<br>ctccagccaagactgatgaacagaaaaaagagtctaagttcctccctttcctcactaatatcgagactctctacaataacctagtgaacaagattgacgactacctgatcaaccttaaagccaagataaacgactgcaatgtcgagaaggatgaggc<br>tcatgttaagatcaccaaactgtccgatctgaaagccatcgacgacaagatcgacttatttaaaaacccatacgatttcgaggctatcaaaaagctgatcaatgatgacaccaagaaagatatgctcggcaagctgctgagcacgggtctggtgcag<br>aacttccctaacaccatcatatcaaagctcatagagggcaagttccaagacatgctgaatatttcacagcatcagtgcgtcaagaagcagtgccccgaaaattctggatgcttccggcacctggatgagcgagaagagtgcaagtgcctgcttaact<br>ataaacaggagggcgacaaatgtgtggagaacccaaatccgacgtgcaacgagaacaacggtggctgcgatgccgacgcgacttgtacagaggaagactcggggagttctcggaaaaaaatcacgtgcgagtgcaccaaacccgacagttatc<br>ctctgttcgatgggatattctgctcctccagcaacgtttag

Supplemental table 2:
Contains the full sequences of synthetic genes used in this study.
